# Reorganization of seagrass communities in a changing climate

**DOI:** 10.1038/s41477-023-01445-6

**Published:** 2023-06-19

**Authors:** Barnabas H. Daru, Brianna M. Rock

**Affiliations:** 1 Department of Biology, Stanford University, Stanford, CA USA; 2 Clearwater Marine Aquarium Research Institute, Clearwater, FL USA

**Keywords:** Ecology, Plant sciences

## Abstract

Although climate change projections indicate significant threats to terrestrial biodiversity, the effects are much more profound and striking in the marine environment. Here we explore how different facets of locally distinctive *α*- and *β*-diversity (changes in spatial composition) of seagrasses will respond to future climate change scenarios across the globe and compare their coverage with the existing network of marine protected areas. By using species distribution modelling and a dated phylogeny, we predict widespread reductions in species’ range sizes that will result in increases in seagrass weighted and phylogenetic endemism. These projected increases of endemism will result in divergent shifts in the spatial composition of *β*-diversity leading to differentiation in some areas and the homogenization of seagrass communities in other regions. Regardless of the climate scenario, the potential hotspots of these projected shifts in seagrass *α*- and *β*-diversity are predicted to occur outside the current network of marine protected areas, providing new priority areas for future conservation planning that incorporate seagrasses. Our findings report responses of species to future climate for a group that is currently under represented in climate change assessments yet crucial in maintaining marine food chains and providing habitat for a wide range of marine biodiversity.

## Abstract (web summary)

Climate change will reduce seagrass ranges leading to increased endemism. Such changes will cause differentiation in some regions and homogenization in other areas. The current marine protected areas will be insufficient to protect seagrasses.

## Main

As Earth’s environment changes at an unprecedented rate^1,2^, it is increasingly recognized that climate change, human exploitation and land- and sea-use changes constitute the major drivers of this change in the modern context^3–7^. These drivers are expected to negatively affect current biodiversity by elevating extinction rates, altering phenologies of species and reshaping ecological communities^1,2,8–11^. Such impacts are most pronounced for marine species (for example, refs. ^2,12–15^) probably because ~80% of the excessive heat from greenhouse emission is directly absorbed by the ocean^16,17^. Along with increasing temperatures and changing ocean chemistry, the frequency of extreme events in coastal areas including wave action, storms and El Niño/Southern Oscillation, also increases^18^. Such events could have dire consequences on the physiology and ecology of marine organisms including foundation species such as seagrasses^19–23^.

Seagrasses are a unique group of basal monocot land plants consisting of ~66–70 species in the order Alismatales that secondarily returned to live in marine habitats ~140 million years ago (Ma). They are widely distributed in coastal and marine environments, providing a range of ecosystem services that rival those of coral reefs and mangroves^24,25^. Seagrasses directly provide food for many marine herbivores including the endangered green sea turtle, manatees and dugongs. Seagrasses form dense meadows that are habitat for many marine invertebrate and vertebrate species and are ideal nursery and foraging grounds for marine fishes and predatory groups, including marine mammals, shorebirds and elasmobranchs^26^. Seagrasses also provide sediment stabilization, carbon sequestration, improvement of water clarity, nutrient uptake and oxygen production^27–29^. However, seagrass meadows worldwide are being lost at an unprecedented rate of 0.9% to 7% per year from anthropogenic impacts such as pollution, nutrient runoff and coastal development^18^. Such losses are perhaps more exacerbated under climate change impacts and can compromise seagrass associated ecosystem goods and services^30^.

Although there is a strong focus on predicting distributional climate-related range shifts for selected seagrass species and at regional scales^31–36^, surprisingly there has never been a global assessment of this phenomenon for seagrasses despite being the only angiosperm group maintaining food chains in the marine environment. Several factors such as scarcity of georeferenced point records, coverage gaps and sampling biases for many regions and clades and lack of analytical tools can constrain research to assess present and future responses of seagrasses to climate change^37^. In the absence of point occurrences, range polygons can be used to model species distributions because they are integrated from point records, expert knowledge of the ecology and distributions of species, local inventories, atlas and literature and yield robust results^38,39^. A global analysis of how seagrasses are responding to climate change is timely and necessary to assess the marine ecosystems that are sensitive to changes in community composition as the Earth system undergoes profound change due to human activity^20^.

In this Article, we explore how predicted range dynamics caused by climate change could contribute to changes in *α*- and *β*-diversity among 66 seagrass species spanning the world’s coastlines. We use species distribution modelling to project the future distributions of seagrasses under various scenarios of climate change. Our definition of *α*-diversity refers to the common diversity indices from the biodiversity literature including species richness (number of species in an area)^40^, phylogenetic diversity (length of branches connecting species from the tip to the root of a phylogenetic tree)^41^, weighted endemism (species richness weighted by range size)^42^ and phylogenetic endemism (range-weighted phylogenetic diversity)^43^. On the other hand, *β*-diversity quantifies the variation in species/phylogenetic composition between sites and/or time^44^. Reductions in *β*-diversity can lead to a phenomenon called spatial homogenization, which results from the simultaneous local disappearance and introduction of new species in a region^45–47^. Specifically, we identify the marine regions that will harbour the greatest evolutionary potential and richness of seagrasses following climate change by comparing species and phylogenetic diversity and composition under current and future climate scenarios based on four different representative concentration pathways (RCP 2.6, 4.5, 6.0 and 8.5) at two time periods, T1 (2040–2050) and T2 (2090–2100). We address three key questions: (1) To what extent will the *α*-diversity (measured as richness and endemism) of seagrass communities change under current and future climate? (2) Will there be universal evidence for shifts in variation in species composition and phylogenetic relatedness between sites under future climate scenarios? and (3) How effective are the global systems of marine protected areas (MPAs) in harbouring future changes in *α*- and *β*-diversity of seagrasses?

Our results project reductions in seagrass species ranges leading to increases in areas of weighted and phylogenetic endemism. Such changes will correspond to gains in *β*-diversity in some regions that will cause seagrass communities to become differentiated but other areas will see regional losses leading to homogenization of seagrass communities under future climate scenarios. We further show that the current network of MPAs will be insufficient to safeguard the future of seagrasses under future climate scenarios.

## Results and discussion

### Changes in species geographic ranges

**Fig. 1:**
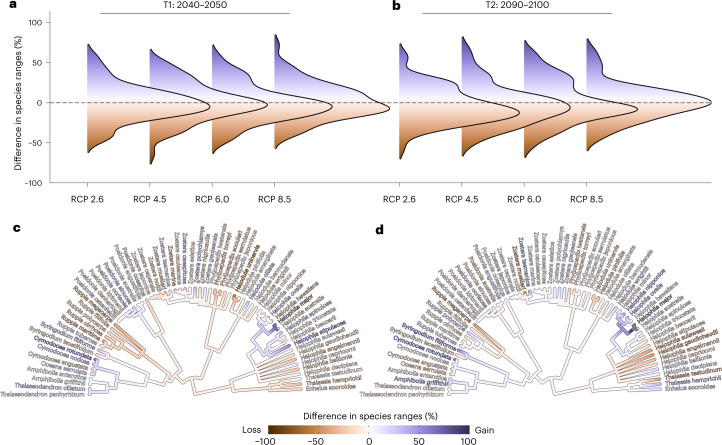
Taxonomic distribution of seagrass species geographic change at two different time horizons. For each species, geographic change was estimated using species distribution models fitted using maximum entropy by calculating the percentage of grid cells either lost or gained through time relative to present day. **a**–**d**, Estimates of species geographic change were visualized using ridgeline density plots (*n* = 66 species) for T1 (2040–2050) (**a**) and T2 (2090–2100) (**b**) under four different RCPs (2.6, 4.5, 6.0 and 8.5); and phylogenetically (*n* = 66 species) for T1 (2040–2050) (**c**) and T2 (2090–2100) (**d**) for RCP 2.6 (see Supplementary Table 1 for geographic change at other RCPs). Negative values indicate reduction in range size and positive values correspond to range expansions. Dashed horizontal lines in **a** and **b** indicate no range change.

For all climate scenarios, our models project widespread range contractions across species with reductions in climatically suitable areas ranging from −3.17% to −0.29% for mid-century 2050 and −6.38% to −0.141% for end-of-century 2100 time horizons (Fig. 1a,b). Of the modelled 66 seagrass species, 31.82% are projected to suffer range losses of >10%. In contrast, 28.79% species will gain ranges of >10%. The magnitude of these changes is weakly and negatively correlated with the range size of species (*r* = −0.2; Spearman rank correlation between current species’ ranges and future range changes; *P* = 0.0000041), indicating that a reduction in seagrass ranges may have a greater impact on the survival of ecologically linked groups in the marine realm with smaller range sizes (manatees, dugongs and marine invertebrates). Our analysis of phylogenetic signal in range changes of species indicates weak support for a correlation between evolutionary relatedness and changes in range size among closely related species, for both mid- and end-of-century time horizons (values of Moran’s *I*, *C*_mean_, Pagel’s *λ* and Blomberg *K* varying from −0.009 to 0.072 all *P* > 0.05; Fig. 1c,d and Supplementary Table 1). This implies that factors beyond shared ancestry, such as environmental changes and anthropogenic impacts, may play a more substantial role in driving range shifts.

### Changes in spatial *α*-diversity

**Fig. 2:**
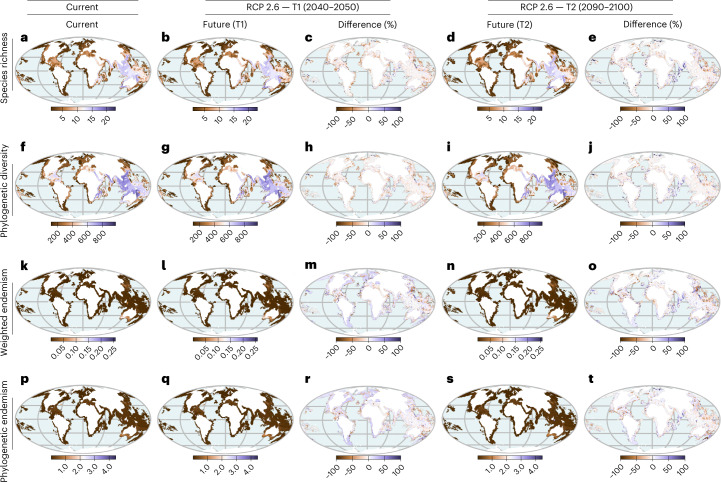
Temporal and geographic patterns of change in *α*-diversity of seagrasses under climate change. Estimates are based on species distribution models of seagrasses (*n* = 66 species) fitted using maximum entropy and aggregated to 100 × 100 km^2^ grid cells. Indicated are the spatial and temporal distributions of: **a–e**, species richness (number of species in a grid cell) for (**a**) current, (**b**) future, and (**c**) difference for mid-century (2050) and (**d**) future, and (**e**) difference forend-of-century (2100); **f–j**, phylogenetic diversity (sum phylogenetic branch lengths connecting species in a grid cell) for (**f**) current, (**g**) future, and (**h**) difference for mid-century (2050) and (**i**) future, and (**j**) difference for end-of-century (2100); and **k–o**, weighted endemism (species richness inversely weighted by species ranges) for (**k**) current, (**l**) future, and (**m**) difference for mid-century (2050) and (**n**) future, and (**o**) difference for end-of-century (2100); **p–t**, phylogenetic endemism (the amount ofevolutionary history that is unique to a particular areas) for (**p**) current, (**q**) future, and (**r**) difference for mid-century (2050) and (**s**) future, and (**t**) difference for end-of-century (2100). Differences in *α*-diversity for each metric are shown for T1 (2040–2050) and T2 (2090–2100) both under RCP 2.6 (best-case scenario). For each difference map (T1 (**c**,**h**,**m**,**r**) and T2 (**e**,**j**,**o**,**t**)), negative values indicate reductions in diversity and positive values correspond to increases in total diversity. Analyses of phylogenetic diversity and phylogenetic endemism were based on a randomly selected subset of 100 trees from a random distribution of 1,000 trees. Projected shifts in *α*-diversity under different climate scenarios are presented in Extended Data Figs. 1–4. The maps are in the Mollweide projection.

**Extended Data Fig. 1:**
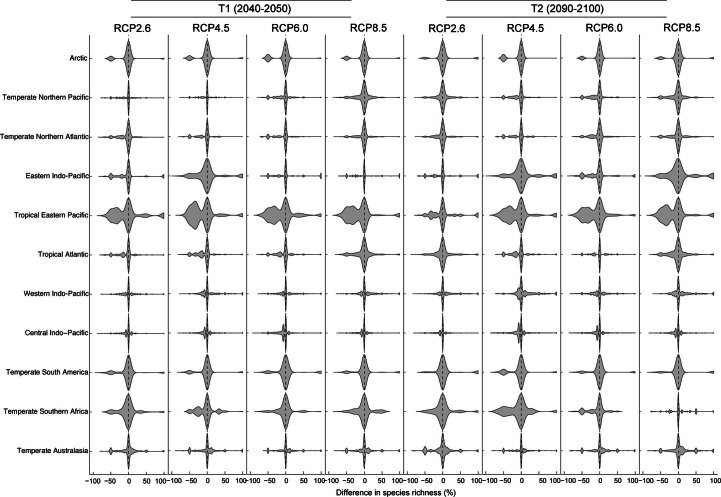
Temporal changes in species richness of seagrasses under scenarios of climate change. Estimates of species richness are based on species distribution models of seagrasses (n = 66 species) fitted using maximum entropy and aggregated as number of species in 100 km × 100 km grid cells. Indicated are the differences in species richness across 11 marine ecoregions for four different representative concentration pathways (RCP2.6, 4.5, 6.0, and 8.5) and for two time periods T1: 2040–2050 and T2: 2090–2100. Negative values indicate reductions in species richness and positive values correspond to increases in richness in a region.

**Extended Data Fig. 2:**
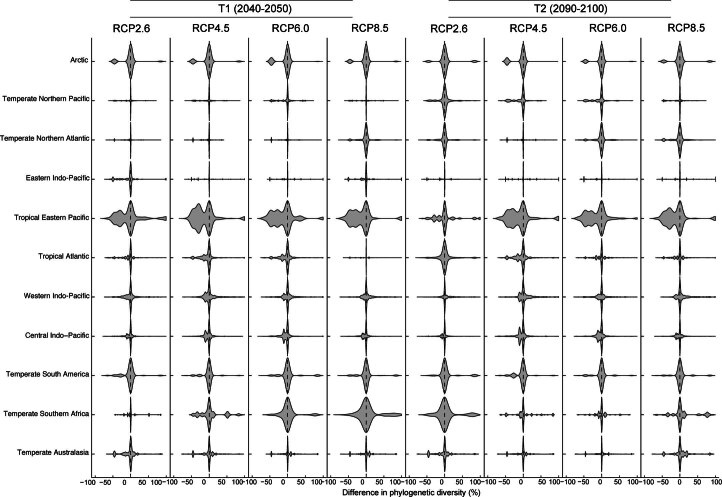
Temporal changes in phylogenetic diversity of seagrasses under scenarios of climate change. Estimates of phylogenetic diversity are based on the species distribution models of seagrasses (n = 66 species) fitted using maximum entropy and aggregated as the sum of phylogenetic branch lengths connecting species in each 100 km × 100 km grid cell. Indicated are the differences in phylogenetic diversity richness across 11 marine ecoregions for four different representative concentration pathways (RCP2.6, 4.5, 6.0, and 8.5) and for two time periods T1: 2040–2050 and T2: 2090–2100. Negative values indicate reductions in phylogenetic diversity and positive values correspond to increases in phylogenetic diversity in a region.

**Extended Data Fig. 3:**
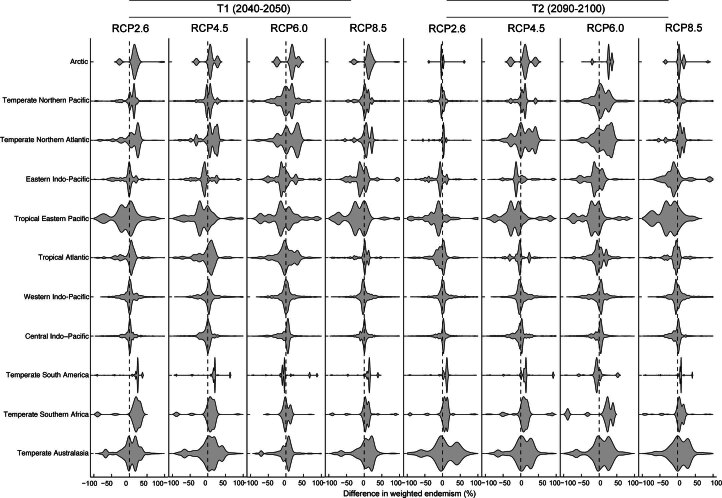
Temporal changes in weighted endemism of seagrasses under scenarios of climate change. Estimates of weighted endemism are based on the species distribution models of seagrasses (n = 66 species) fitted using maximum entropy and aggregated as species richness inversely weighted by species ranges in each 100 km × 100 km grid cell. Indicated are the differences in weighted endemism across 11 marine ecoregions for four different representative concentration pathways (RCP2.6, 4.5, 6.0, and 8.5) and for two time periods T1: 2040–2050 and T2: 2090–2100. Negative values indicate reductions in weighted endemism and positive values correspond to increases in weighted endemism in a region.

**Extended Data Fig. 4:**
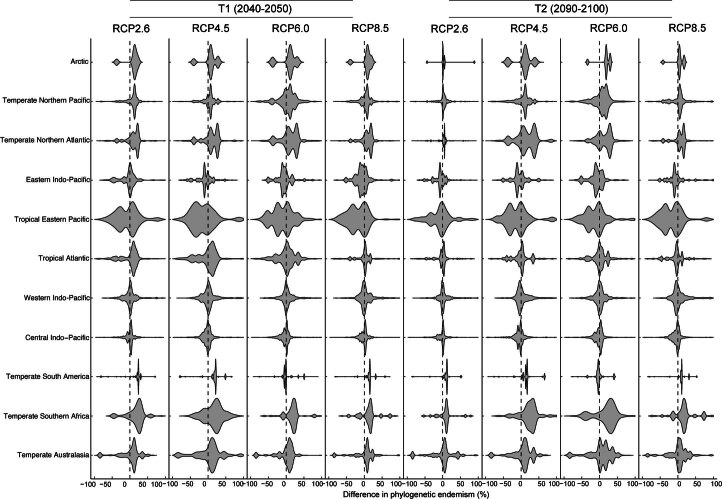
Temporal changes in phylogenetic endemism of seagrasses under scenarios of climate change. Estimates of phylogenetic endemism are based on the species distribution models of seagrasses (n = 66 species) fitted using maximum entropy and aggregated as phylogenetic diversity restricted to any 100 km × 100 km grid cell. Indicated are the differences in phylogenetic endemism across 11 marine ecoregions for four different representative concentration pathways (RCP2.6, 4.5, 6.0, and 8.5) and for two time periods T1: 2040–2050 and T2: 2090–2100. Negative values indicate reductions in phylogenetic endemism and positive values correspond to increases in phylogenetic endemism in a region.

Using four different future climate RCPs at two different time horizons (2050 and 2100), we evaluate changes in seagrass *α*-diversity by quantifying differences in species richness, phylogenetic diversity, species weighted endemism and phylogenetic endemism under current and future climate scenarios. Our maps of species richness and phylogenetic *α*-diversity based on the current climate align well with future richness maps by 2050 and 2100 (Fig. 2), indicating no notable changes in *α*-diversity metrics between current and projected distributions, except in the Tropical Eastern Pacific and Central Indo-Pacific, where losses in species richness and phylogenetic *α*-diversity will be more pronounced (Fig. 2 and Extended Data Figs. 1–4). Most areas of seagrass diversity are projected to experience increases in weighted endemism and phylogenetic endemism (Fig. 2), supporting the projected decrease in range size for most species (Fig. 1), except for the Tropical Eastern Pacific and Eastern Indo-Pacific where losses in endemism will be substantial. Areas of endemism are crucial for conservation due to their unique and irreplaceable biodiversity, including species that are found nowhere else in the world and have no close relatives in other regions^43,48,49^. Spatial correlations of current and future changes of these facets of *α*-diversity revealed a weak to negligible relationship that was consistent across different grain resolutions (*r*_*s*_ = −0.19 to 0.049; Supplementary Table 2). This suggests that the drivers of current *α*-diversity may not be the same as those that will drive future changes in *α*-diversity but rather reflect fundamental ecological processes that operate at multiple scales. The trend of increasing endemism (places with high concentrations of range-restricted species and phylogenetic branch lengths) is projected to be similar for both mid-century (2050) and end-of-century (2100) scenarios, suggesting that environmental conditions will become unfavourable for seagrasses under future climate change with repercussions for conservation planning^22,50^. These findings are in general agreement with well-known patterns of the responses of marine species to climate change^4,13,14,17,51^, where climatically suitable areas are reduced under worsening climate scenarios, thus leading to increased endemism in the future^52,53^. Not surprisingly, these global findings may hide regional variation in seagrass communities where changes in future species and phylogenetic composition might be more profound.

### Changes in *β*-diversity

**Fig. 3:**
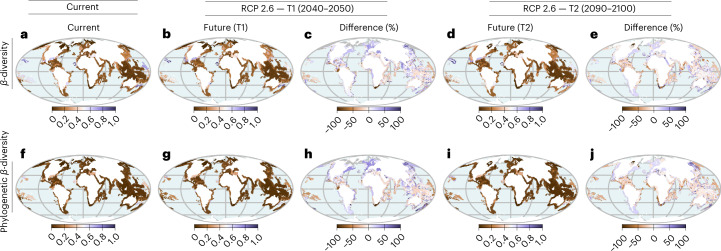
Geographic and temporal changes in *β*-diversity in seagrasses under climate change. **a–e**, Spatial and temporal changes in species *β* diversity for (a) current, (b) future, and (c) difference for mid-century (2050) and (**d**) future, and (**e**) difference for end-of-century (2100). **f–j**, Spatial and temporal changes in phylogenetic *β* diversity for (**f**) current, (**g**) future, and (**h**) difference for mid-century (2050) and (**i**) future, and (**j**) difference for end-of-century (2100). Changes in *β*-diversity were based on species distribution models fitted using maximum entropy and estimated using Simpson dissimilarity index for grid cells across time. Differences in *β*-diversity for each metric are shown for T1 (2040–2050) and T2 (2090–2100) both under RCP 2.6 (best-case scenario). Positive values in **c**, **e**, **h** and **j** indicate increasing dissimilarity (differentiation) and negative values correspond to decreasing dissimilarity (homogenization). Temporal and spatial changes in *β*-diversity were calculated across marine ecoregions of the world. The maps are in the Mollweide projection.

**Extended Data Fig. 5:**
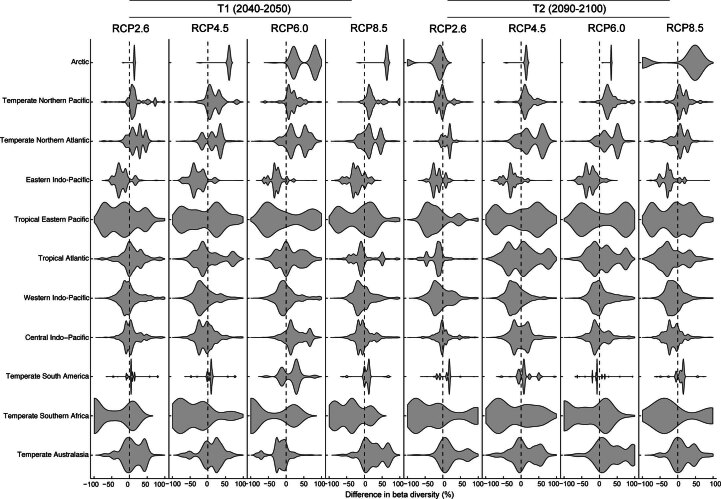
Temporal changes in species beta diversity of seagrasses under scenarios of climate change. Estimates of beta diversity are based on the species distribution models of seagrasses (n = 66 species) fitted using maximum entropy and aggregated as the variation in species composition between sites that is, between 100 km × 100 km grid cells. Indicated are the differences in beta diversity across 11 marine ecoregions for four different representative concentration pathways (RCP2.6, 4.5, 6.0, and 8.5) and for two time periods T1: 2040–2050 and T2: 2090–2100. Negative values indicate reductions in beta diversity (that is, species composition will become more identical) and positive values correspond to increases in beta diversity (that is, species composition will become more dissimilar) in the future.

**Extended Data Fig. 6:**
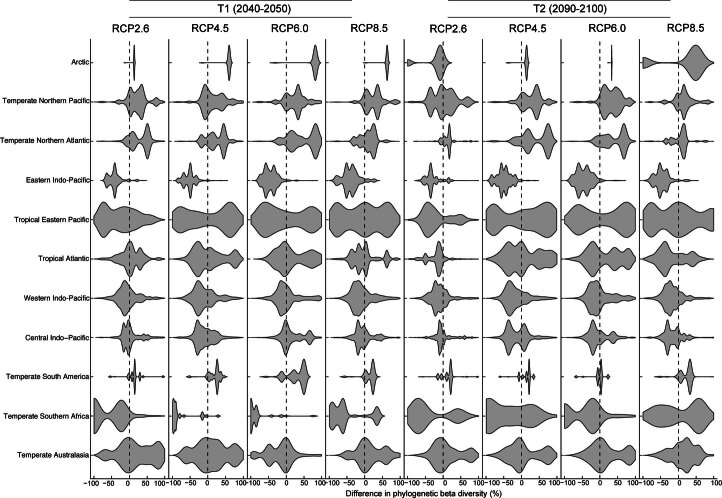
Temporal changes in phylogenetic beta diversity of seagrasses under scenarios of climate change. Estimates of phylogenetic beta diversity are based on the species distribution models of seagrasses (n = 66 species) fitted using maximum entropy and aggregated as the variation in phylogenetic composition between sites that is, between 100 km × 100 km grid cells. Indicated are the differences in phylogenetic beta diversity across 11 marine ecoregions for four different representative concentration pathways (RCP2.6, 4.5, 6.0, and 8.5) and for two time periods T1: 2040–2050 and T2: 2090–2100. Negative values indicate reductions in phylogenetic beta diversity (that is, phylogenetic composition will become more identical) and positive values correspond to increases in beta diversity (species composition will become more dissimilar) in the future.

To identify aspects of biodiversity change that are decoupled from species richness and phylogenetic diversity, we mapped changes in *β*-diversity (the spatial composition of species and phylogenetic diversity between local communities) under various climate scenarios. Our models project gains in species and phylogenetic *β*-diversity across most regions, ultimately causing seagrass communities to become increasingly differentiated by 2050 and 2100 (Fig. 3). The opposite is true, however, across marine ecoregions of the Eastern Indo-Pacific, Tropical Eastern Pacific, Western Indo-Pacific and Temperate Southern Africa where *β*-diversity of species and phylogenetic diversity are projected to decrease leading to homogenization of seagrasses in these regions (Fig. 3 and Extended Data Fig. 5 and 6). These findings support the hypothesis that dispersal limitation (species with projected range reductions) can cause *β*-diversity to increase, resulting in greater differentiation across regions, whereas the composition of species connected by high dispersal rates will be more homogenous across geographic space^54^. The regions of projected increases in *β*-diversity could correspond to regions where closely related species such as *Ruppia* and *Halophila* are predicted to show range contractions (become more endemic) in the future. These areas are also heavily impacted by human activities such as overfishing, invasion and pollution^30,55,56^. Such results could imply that transient species may be occupying regions where they did not previously exist, yielding an overall shift in *β*-diversity across regions. Our projections of climate change causing divergent shifts in seagrass *β*-diversity—differentiation in some regions and homogenization in other regions—are robust to varying assumptions of climate scenarios, grain resolutions (50, 100, 200, 400 and 800 km grain sizes) and current diversity patterns (Supplementary Table 2).

Our analysis of climate impacts on seagrasses is in general agreement with previous findings that environmental factors such as sea temperature, salinity and water depth represent the most essential factors in explaining spatial variation of seagrass diversity^18,30–33^. Such potential shifts in distributions may have profound implications for seagrasses, marine photosynthetic activity and associated fauna. For instance, our models suggest that as seagrass ranges expand, some species may colonize deeper areas with lower light availability, potentially reducing overall primary productivity in these light-limited zones^57^. Likewise, negative impacts of climate change on seagrasses, being foundational species, may translate to breakdown of biotic interactions of other ecologically connected taxonomic groups potentially increasing their vulnerability to ecological and evolutionary perturbations and, ultimately, extinction^58–60^. Thus, optimizing the protection of seagrasses can help safeguard and increase the diversity of other ecologically linked groups^58,61^.

### MPA and future of seagrass biodiversity

To assess how well future hotspots of seagrasses will be protected by MPAs, we calculated the proportions of the seascape in each grid cell that fall within MPAs of the International Union for Conservation of Nature (IUCN) categories I–IV^62^. Hotspot cells were identified using the 2.5% threshold corresponding to 97.5th percentile values for each diversity metric relative to present day; a common approach in macroecology^63,64^.

**Fig. 4:**
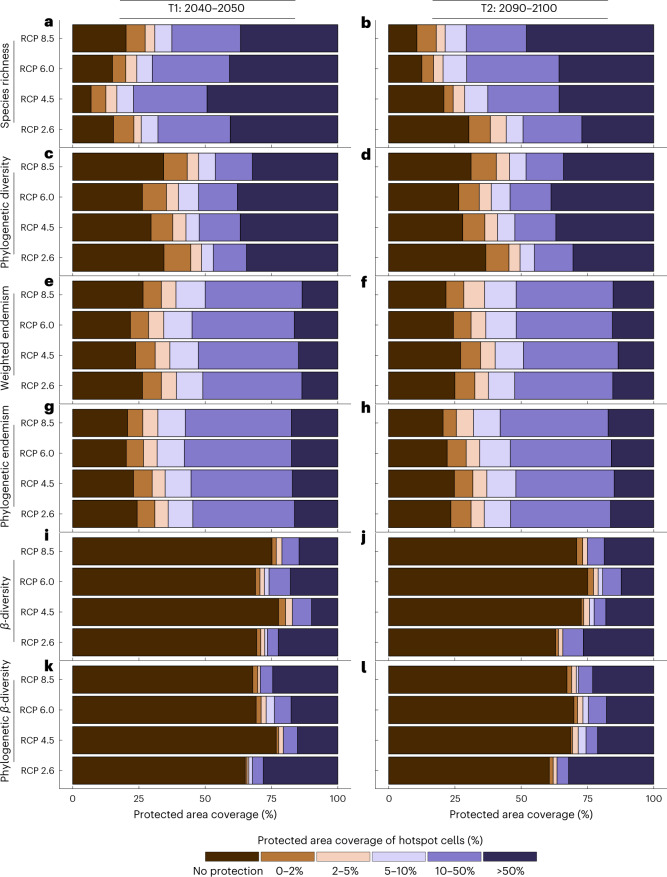
Overlap of MPAs with future hotspots of *α*- and *β*-diversity of seagrasses. **a**–**l**, Indicated are overlaps with hotspots of: species richness (**a**,**b**), phylogenetic diversity (**c**,**d**), weighted endemism (**e**,**f**), phylogenetic endemism (**g**,**h**), *β*-diversity (**i**,**j**) and phylogenetic *β*-diversity (**k**,**l**). Overlaps are shown for future hotspots in T1 (2040–2050) (**a**,**c**,**e**,**g**,**i**,**k**) and T2 (2090–2100) (**b**,**d**,**f**,**h**,**j**,**l**) across four different RCPs (2.6, 4.5, 6.0 and 8.5). Overall, only a modest fraction of future hotspots will be contained within MPAs. Analyses of phylogenetic diversity and phylogenetic endemism were based on a randomly selected subset of 100 trees from a random distribution of 1,000 trees.

Our results show that while some future hotspots of *α*-diversity will be protected by MPAs, a significant proportion will not (Fig. 4). This proportion is projected to increase over time. By mid-century, the percentage of hotspots not protected will range from 6.95% for species richness to 26.26% for phylogenetic diversity. By the end of the century, these figures are projected to increase to 30.28% and 36.67%, respectively (Fig. 4a–d). Similarly, the proportion of future weighted endemism and phylogenetic endemism hotspots not included within MPAs is expected to range from 21.76% to 27.15% and 20.21% to 24.83%, respectively (Fig. 4e–h). In terms of metrics of compositional turnover, we found that a significant proportion of *β*-diversity hotspots, when compared to their phylogenetic counterparts, will probably fall outside MPAs in the future (Fig. 4i–l). Compositional turnover is a key component of *β*-diversity, which quantifies the rate species change along environmental gradients or between habitats^65^. High rates of turnover in *β*-diversity hotspots can indicate the presence of unique ecological niches and habitats that harbour a high diversity of species^63,66^. Our results indicate that between mid-century and the end of the century, 68.94% to 75.11% of future *β*-diversity hotspots will remain unprotected by MPAs, while 65.58% to 69.86% of future phylogenetic *β*-diversity hotspots will not be covered by these conservation measures. The *β*-diversity hotspots are critical for the maintenance of seagrass ecosystems and their associated biodiversity, as these areas capture high levels of diversity and support the sustenance of diverse ecological communities.

These findings indicate that the current network of MPAs will be insufficient to safeguard future hotspots of seagrass diversity, supporting evidence from analyses of coral reefs^67^, sea turtles^68^ and marine fish species diversity^69^. Such limitations of the MPAs in safeguarding seagrass diversity in the future may indicate that MPAs were originally designed to conserve animal resources, including fish stocks and coral reefs^70^. However, there has been increasing recognition of seagrass and coastal ecosystems for conservation and ecosystem services, like nutrient cycling and carbon storage, resulting in the expansion of the scope of MPAs to protect seagrass biodiversity and ecosystem services (for example, refs. ^71,72^). The highlighted unprotected hotspots provide new priority areas for planning future conservation actions to better incorporate seagrasses, particularly in regions such as the East China Sea, the Great Australian Bight, Southern China, the South China Sea Oceanic Islands, the Gulf of Tonkin, the Yellow Sea and the Sulawesi Sea/Makassar Strait. These regions have high levels of species richness, phylogenetic diversity and endemism (Supplementary Tables 3–6), making them crucial for maintaining global biodiversity and ecosystem services such as nutrient cycling and carbon storage. In addition, focusing conservation efforts on unprotected hotspots of species and phylogenetic *β*-diversity in mid-century and end-of-century, such as Cape Verde, Hawaii, Lord Howe and Norfolk Islands and Northern Gulf of Mexico, could better protect seagrasses and diverse ecological communities (Supplementary Tables 7 and 8).

**Extended Data Fig. 7:**
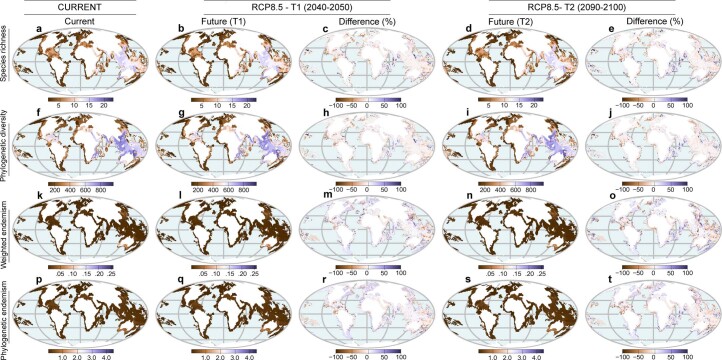
Temporal and geographic patterns of change in alpha diversity of seagrasses under a worst-case scenario of climate change. Estimates are based on species distribution models of seagrasses (n = 66 species) fitted using maximum entropy and aggregated to 100 km × 100 km grid cells. Indicated are the spatial and temporal changes between current and future distributions of α-diversity based on: a–e, species richness (number of species in a grid cell), f–j, phylogenetic diversity (sum of phylogenetic branch lengths connecting species in a grid cell), k–o, weighted endemism (species richness inversely weighted by species ranges), and, p–t, phylogenetic endemism (the amount of evolutionary history that is unique to a particular area). Differences in α-diversity for each metric are shown for T1: 2040–2050 and T2: 2090–2100 both under RCP8.5 (worst-case scenario). For each difference map (T1: c, h, m, r, and T2: e, j, o, t), negative values indicate reductions in diversity and positive values correspond to increases in total diversity. Analyses of phylogenetic diversity and phylogenetic endemism were based on a randomly selected subset of 100 trees from a random distribution of 1000 trees.

**Extended Data Fig. 8:**
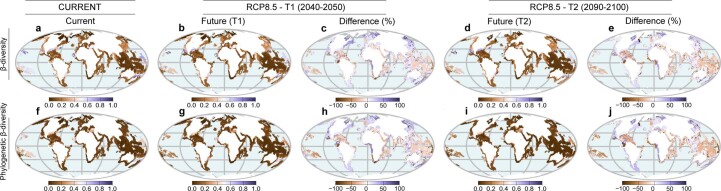
Geographic and temporal changes in beta diversity in seagrasses under a worst-case scenario of climate change. a–e Spatial and temporal changes in species (β) diversity. f–j Spatial and temporal changes in phylogenetic (β) diversity. Changes in beta diversity were based on species distribution models fitted using maximum entropy and estimated using Simpson dissimilarity index for grid cells across time. Differences in β-diversity for each metric are shown for T1: 2040–2050 and T2: 2090–2100 both under RCP8.5 (worst-case scenario). Positive values in c, e, h, and j indicate increasing dissimilarity (differentiation) and negative values correspond to decreasing dissimilarity (that is, homogenization). Temporal and spatial changes in beta diversity were calculated across marine ecoregions of the world. The maps are in the Mollweide projection.

Our analyses used IUCN polygons to model present and future distributions of seagrasses under climate scenarios. Although these are broad-scale approximations of species distributions with underlying assumptions that all populations within a species have similar environmental requirements^73^, our results are consistent across climate scenarios (Extended Data Figs. 7 and 8) and grain sizes. Our results are also robust to the addition of phylogenetic information and yielded strong model performance scores based on true skill statistics (TSS) and area under the receiver operator curve (AUC) scores.

In conclusion, we project widespread range contractions and increases in areas of weighted and phylogenetic endemism for seagrasses with the goal of highlighting priority areas for future conservation planning. These shifts will translate into gains in *β*-diversity in some regions that will cause seagrass communities to become increasingly differentiated but other areas will see regional losses resulting in the homogenization of seagrass communities under future climate scenarios. The hotspots of these projected shifts in seagrass *α*- and *β*-diversity are predicted to occur outside the current network of MPAs, implying that these MPAs will be insufficient to preserve seagrasses into the future. Given that these trends are similar across climate scenarios, our analysis suggests that the response of marine primary producers to future climate change is consistent and potentially predictable.

## Methods

### Species occurrence data and taxonomic harmonization

Occurrence data of seagrasses were collated from public open-source databases: the Global Biodiversity Information Facility (GBIF; https://www.gbif.org/), Seagrass Watch (www.seagrasswatch.org), iDigBio (https://www.idigbio.org/), Ocean Biogeographic Information System (OBIS; https://obis.org/) and the IUCN. The criteria for including a record were that it should capture information on the species name, geographic location and collection date and a species should have at least 25 unique records. However, we previously demonstrated that occurrence records of seagrasses are very scarce and prevalent with coverage gaps and biases^37^ and thus can constrain research to assess present and future seagrass response from climate change^37^. Therefore, we used range polygons from the IUCN^74^ to model present and future distributions of seagrasses under climate scenarios. We assume that this method is valid because of the strong agreement between occurrence data and range polygons in producing highly similar species distribution model outputs in other taxonomic groups^38,39,75–77^. Along these lines, we converted the species polygons into raster format at a grain resolution of 5 arcmin (~9.2 km) and then to points and treat them as real point occurrence records. For each species, we spatially thinned occurrences to 500 records to avoid spatial bias in the modelling^77^. We then standardized the taxonomy of each seagrass species by checking for misspellings, synonyms, formatting errors, hybrid names and infraspecific ranks, against the backbone taxonomy from the World Flora Online v.2022.05 (ref. ^78^). Taxonomic harmonization was done using the R package WorldFlora^79^ and manually checked in cases of misspellings or errors. The final checklist included 66 valid species which were all included in the analysis.

### Current and future climate dataset

Current and global future climate layers were sourced from the Bio-ORACLE v.2.2 database (https://www.bio-oracle.org/) at a spatial grain resolution of 5 arcmin (~9.2 km). These variables were downloaded for four RCPs (2.6, 4.5, 6.0 and 8.5) and are derived from three atmosphere–ocean general circulation models (AOGCMs) and retrieved from Bio-ORACLE v.2.2 datasets^80,81^. The variables for the future scenarios were obtained for two time periods—T1 2050 (2040–2050) and T2 2100 (2090–2100)—representing medium and long terms, respectively. We refer to RCP 2.6 as best-case scenario because it is representative of a peak-and-decline scenario ending with very low greenhouse gas concentration levels by the end of the twenty-first century, whereas RCP 4.5 and RCP 6.0 are stabilized scenarios in which concentration levels stabilize; and RCP 8.5 as worst case because it is representative of a scenario of increasing emissions over time leading to high greenhouse gas concentration levels^80,81^. The variables for both current and future scenarios included in the modelling efforts were annual mean and range surface and benthic sea temperature, salinity and currents velocity. These variables were selected because they are significant for seagrass growth, distribution and photosynthesis^82^.

### Species distribution modelling

The species distribution models follow the ODMAP (overview, data, model, assessment, prediction) protocol for reporting species distribution models^83^. The objective for the species distribution model was to predict seagrass species occurrences in space as binary maps of potential presences. These maps were stacked and converted to a community matrix for downstream analyses such as predicting species assemblages.

We defined a migration limit for each species by intersecting a 4° buffer around species occurrences and the marine ecoregions occupied by the species^84^. This limit represents the attainable distance of dispersal and ecological limitation for most species. Our approach is commonly used in distribution modelling of other taxonomic groups, such as terrestrial amphibians, birds, mammals, reptiles, lycophytes, flowering plants, ferns and gymnosperms^39,85^. By considering these taxonomic groups, we can make more informed decisions about the migration limits of seagrasses, which are typically dispersed over similar distances^86^. We used the observed presences as input with a 75% random sample for model development and the remaining 25% sample for model evaluation. Species distribution models were fitted using maximum entropy (MaxEnt) using the function sdm in the R package phyloregion^64^ and applied to the observed species occurrences and climatic variables for both current and future scenarios. We used MaxEnt because it does not create response curves that may cause unpredictable behaviour when applied to new climates^87^. Model settings were chosen to yield intermediate complex response surfaces. For each species, we selected 10,000 pseudo-absences within the model calibration area as background points. We used the equal training sensitivity (true positive rate) and specificity threshold (true negative rate)^88^ to convert the continuous predicted probabilities into binary presence–absence maps. Model performances were evaluated using the AUC^89^ and TSS^90^ scores. TSS scores range from −1 to 1 whereas AUC scores range from 0 (prediction of absence) to a maximum of 1 (predicted presence)^89,90^ with the threshold of good performing models within the 2.5–97.5 percentile range as is common practice^39,87,90,91^.

For each species, the model prediction consisted of a range map stored in raster format at 5 arcmin grid cell resolution. To represent the species distribution as a continuous surface analogous to the mapping process used by the IUCN, we dissolved each spatial raster to polygon using the R package terra^92^. In the final step, we smoothed the jagged edges and sharp corners of the polygon maps to appear more natural by using spline interpolation of vertices in the R package smoothr^93^. All calculations were processed in parallel using open-source software on the Sherlock High Performance Computing clusters of Stanford University. This was achieved by splitting the global point occurrence records into random groups of five species and each sent to single computational node.

The predicted distributions were converted to a community matrix by intersecting with equal-area grid cells at five different grain resolutions: 50, 100, 200, 400 and 800 km using the function polys2comm in the R package phyloregion^64^ for downstream analysis.

### Phylogenetic data

The phylogenetic tree used here was estimated using Bayesian analysis of 66 species and 3,738 base pairs of DNA sequences derived from a combination of *rbcL*, ITS and 18S, assuming an uncorrelated relaxed molecular clock model, using the programme BEAST v.1.7.5 (ref. ^94^). Branch lengths were calibrated in millions of years using a Bayesian MCMC approach by enforcing topological constraints assuming APG III backbone from Phylomatic v.3 (ref. ^95^) and six fossil calibration points from the literature: Alismatales crown node 128 Ma, Cymodoceae crown node 61 Ma, Zosteraceae crown node 17 Ma, Hydrocharitaceae crown node 75 Ma and Tofieldiaceae crown node 100 Ma^96^ and Alocasia crown node 19.28 Ma^97^. Full details of the phylogeny reconstruction are provided in ref. ^98^.

### Taxonomic distribution of species geographic change

Species geographic change was assessed by converting the continuous predicted probabilities (the raster layers) into binary presence–absence maps. For each species, geographic change was assessed as the difference between the number of pixels with climate suitability value of 1 in the present versus future scenarios divided by the present. This was then standardized as percentage to make it straightforward to interpret change. We tested for phylogenetic signal in the tendency of closely related species having similar geographic change for each climate scenario using four methods that are most widely used in macroecology: Moran’s *I*^99^, Abouheif’s *C*_mean_^100^, Pagel’s lambda (*λ*)^101^ and Blomberg’s *K* (ref. ^102^). Values of Moran’s *I*, *C*_mean_, *λ* and *K* have an expected score of 1 if close relatives share similar geographic change. We repeated this process 1,000 times to assess statistical significance.

### Spatial changes in *α*-diversity

Changes in *α*-diversity were determined by separately computing species richness (SR), weighted endemism (WE), phylogenetic diversity (PD) and phylogenetic endemism (PE) between current and future climate scenarios. For each grid cell, change for each metric of *α*-diversity was calculated as the difference between *α*-diversity in the future and the present and standardized as a percentage:

$$\alpha =\frac{{\alpha }_{j}-{\alpha }_{i}}{{\alpha }_{i}}\,\times \,100 \%$$

where *i* is *α*-diversity under current climate and *j* is *α*-diversity under future scenarios. Species richness is defined as the number of species represented in an ecological community, seascape or region and was calculated as the observed number of species within a grid cell^40^. Phylogenetic diversity is measured as the sum of phylogenetic branch lengths spanning from the tips to the root of a dated phylogenetic tree^41^. WE is defined as the sum of the number of species present in each cell and was determined as species richness inversely weighted by species ranges^42^. WE was computed using the weighted_endemism function in the R package phyloregion^64^. PE is defined as the total phylogenetic diversity spanned by species in a region and was calculated by dividing each unit of phylogenetic diversity by the range size of its extant descendant clade^43^. By using branch length information of descendant clades, PE allows us to measure the spatial restriction of phylogenetic diversity in Myr km^−2^. Calculation of PE was done using the function phylo_endemism also in the R package phyloregion^64^ and is expressed as:

$${\mathrm{PE}}=\sum _{\{i\in I\}}\frac{{L}_{i}}{{R}_{i}}$$

where {*I*} indicates the phylogenetic branches of species spanning from the tip to the root of a dated phylogenetic tree, *L*_*i*_ is the length of phylogenetic branch *i*, calculated as proportion of the total length of the tree and *R*_*i*_ is the geographic range of the clade. For each metric, negative values indicate reductions and positive values correspond to increases in total diversity. For instance, a change of +2% in species richness means projected increase in richness by 2% in future and a change of −2% in weighted endemism correspond to projected loss of weighted endemism (species becoming more widespread) by 2% in future and so on. We accounted for phylogenetic uncertainty by using the multidi function in the R package ape^103^ to resolve polytomies randomly. We ran each analysis across 100 trees and obtained a median result.

### Changes in spatial composition of *β*-diversity

To quantify turnover in species identities that are different from species richness and phylogenetic diversity, we mapped *β*-diversity (the spatial composition species and phylogenetic composition between local communities) under climate scenarios. Within marine ecoregions of the world^84^, pairwise distance matrices of phylogenetic *β*-diversity and species level *β*-diversity were generated between all pairs of grid cells in each marine ecoregion for current and future times. We used the Simpson index, *β*_sim_, which measures the differences in species composition between two sites, to represent our results because it is independent of species richness across sites^44,104^ and, therefore, provides a reliable estimate of changes in community composition. Values of *β*_sim_ vary between 0 (species/phylogenetic composition is identical between grid cells) and 1 (complete turnover, no shared taxa). Thus, changes in *β*-diversity were calculated for each grid cell within a marine ecoregion as the difference between diversity in the future versus present and expressed as a percentage:

$$\beta =\,\frac{{\beta }_{j}\,-\,{\beta }_{i}}{{\beta }_{i}}\,\times \,100 \%$$

where *i* is species composition under current climate and *j* is species composition under future scenarios. Phylogenetic *β*-diversity was calculated using the function phylobeta(*x*, phy) in the R package phyloregion^64^, where *x* is a community composition object of class Matrix or matrix and phy is a phylogenetic tree of the class phylo. The *β*-diversity was calculated using phyloregion’s function beta_diss(*x*), where *x* is a community composition object of class Matrix or matrix. Within geographic regions, we calculated percentage changes in *β*-diversity between pairs of grid cells and mapped these values to explore spatial and temporal changes in compositional turnover across seagrass communities. Percentage change in *β*-diversity close to zero indicates no change in *β*-diversity through time, negative values indicate reductions in *β*-diversity that can result in communities becoming more homogenized, whereas positive values correspond to increases in *β*-diversity leading to differentiation across seagrass communities.

### MPA network analysis

To assess if the current network of MPAs will ensure future distribution of seagrasses, we mapped potential hotspots of *α*- and *β*-diversity. Hotspots were defined as areas with high density of each metric, using the 97.5th percentile values for each diversity metric relative to present day^63,64^. Metrics of *β*-diversity represent areas with high compositional turnover and ecological differentiation. We used the most updated version of the MPAs of the IUCN categories I–IV compiled from the World Database on Protected Areas (WDPA)^62^. Next, we overlaid each potential hotspot onto the MPAs and computed the proportion of cell overlapping with the network of MPAs using the erase function in the R package terra^92^. For future hotspots that are not covered by any MPAs, we identified priority areas by overlapping them with marine ecoregions of the world. See ‘Data availability’ to access the data and analysis codes^105^.

### Reporting summary

Further information on research design is available in the Nature Portfolio Reporting Summary linked to this article.

### Supplementary information

Supplementary information Supplementary tables 1–8.

Reporting Summary

## Data Availability

All data and codes necessary to repeat the analyses described here have been made available through FigShare (10.6084/m9.figshare.21905826).

## Code availability

All scripts, codes and data documentation necessary to repeat our analyses have been made available through FigShare (10.6084/m9.figshare.21905826) under the folder ‘SCRIPTS’.
