# Supplementary material for: Reorganization of seagrass communities in a changing climate
Source: Nat Plants. 2023 Jun 19;9(7):1034–43. doi: 10.1038/s41477-023-01445-6 (PMC10356593; doi:10.1038/s41477-023-01445-6)
Supplement: Supplementary file 1 — Supplementary tables 1–8. [file 41477_2023_1445_MOESM1_ESM.pdf]

nature plants

Article

https://doi.org/10.1038/s41477-023-01445-6

Reorganization of seagrass communities in a changing climate

In the format provided by the authors and unedited

Table of Contents:

Supplementary Tables 1 to 8

Supplementary Table 1. Tests of phylogenetic signal in species geographic change under climate scenarios. All tests are based on 1000 randomizations whereby the trait values are randomly shuffled across the phylogenetic tree multiple times to create null distributions, and the observed value is compared to the null distributions to determine the p-value.

	(a) T1: 2040-2050				(b) T2: 2090-2100			
**Phylogenetic signal test**	**RCP2.6**	**RCP4.5**	**RCP6.0**	**RCP8.5**	**RCP2.6**	**RCP4.5**	**RCP6.0**	**RCP8.5**
Moran's I	-0.0016 (p = 0.39)	-0.0033(p = 0.4)	0.013 (p = 0.36)	0.064 (p = 0.14)	-0.009 (p = 0.47)	-0.014 (p = 0.46)	-0.047 (p = 0.66)	-0.086 (p = 0.9)
Abouheif's C_{mean}	0.0063 (p = 0.44)	0.02 (p = 0.4)	0.025 (p = 0.37)	0.072 (p = 0.14)	0.0012 (p = 0.47)	-0.0052 (p = 0.53)	-0.036 (p = 0.68)	-0.076 (p = 0.92)
Pagel's λ	0.000066 (p = 1)	0.000066 (p = 1)	0.000066 (p = 1)	0.034 (p = 0.62)	0.000066 (p = 1)	0.026 (p = 0.62)	0.049 (p = 0.49)	0.000066 (p = 1)
Blomberg's K	0.034 (p = 0.5)	0.056 (p = 0.082)	0.044 (p = 0.15)	0.027 (p = 0.67)	0.039 (p = 0.53)	0.034 (p = 0.59)	0.044 (p = 0.2)	0.046 (p = 0.41)

Supplementary Table 2. Spatial correlations current and future changes in six diversity metrics. We present results for five spatial grains, 50, 100, 200, 400, and 800 km. For each diversity metric (species richness, phylogenetic diversity, weighted endemism, phylogenetic endemism, beta diversity and phylogenetic beta diversity) current pattern was contrasted to future changes in the diversity metric using a modified t-test which assesses correlation between two spatial processes. The Spearman's rank coefficient (r_s) was used as the statistical test, and no adjustments were made for multiple comparisons. The exact p-values are indicated in parentheses.

		(a) T1: 2040-2050				(b) T2: 2090-2100			
	**Grain (km)**	**RCP2.6**	**RCP4.5**	**RCP6.0**	**RCP8.5**	**RCP2.6**	**RCP4.5**	**RCP6.0**	**RCP8.5**
**Species richness**									
	50	-0.058 (p = 0.16)	-0.14 (p = 0.048)	-0.09 (p = 0.0048)	-0.087 (p = 0.051)	-0.17 (p = 0.0006)	-0.12 (p = 0.057)	-0.11 (p = 0)	-0.17 (p = 0.0026)
	100	-0.041 (p = 0.29)	-0.1182 (p = 0.087)	-0.083 (p = 0.016)	-0.08 (p = 0.075)	-0.16 (p = 0.0004)	-0.11 (p = 0.065)	-0.091 (p = 0)	-0.16 (p = 0.001)
	200	-0.011 (p = 0.8)	-0.09 (p = 0.24)	-0.08 (p = 0.035)	-0.073 (p = 0.15)	-0.14 (p = 0.0001)	-0.11 (p = 0.063)	-0.078 (p = 0.0023)	-0.14 (p = 0.0024)
	400	0.0112 (p = 0.87)	-0.072 (p = 0.39)	-0.076 (p = 0.055)	-0.061 (p = 0.26)	-0.12 (p = 0.0001)	-0.12 (p = 0.076)	-0.089 (p = 0.017)	-0.13 (p = 0.0097)
	800	0.034 (p = 0.59)	-0.077 (p = 0.31)	-0.032 (p = 0.54)	-0.01 (p = 0.89)	-0.12 (p = 0.028)	-0.058 (p = 0.54)	-0.095 (p = 0.06)	-0.051 (p = 0.47)
**Phylogenetic diversity**									
	50	-0.092 (p = 0)	-0.17 (p = 0.0029)	-0.11 (p = 0.0001)	-0.13 (p = 0.0004)	-0.19 (p = 0)	-0.13 (p = 0.02)	-0.13 (p = 0)	-0.18 (p = 0.0012)
	100	-0.078 (p = 0)	-0.14 (p = 0.011)	-0.11 (p = 0.0004)	-0.12 (p = 0.0012)	-0.17 (p = 0)	-0.12 (p = 0.02)	-0.11 (p = 0)	-0.17 (p = 0.0004)
	200	-0.052 (p = 0.036)	-0.11 (p = 0.07)	-0.095 (p = 0.002)	-0.11 (p = 0.013)	-0.16 (p = 0)	-0.12 (p = 0.012)	-0.10 (p = 0.0003)	-0.14 (p = 0.002)
	400	-0.041 (p = 0.42)	-0.094 (p = 0.23)	-0.094 (p = 0.015)	-0.092 (p = 0.042)	-0.16 (p = 0)	-0.13 (p = 0.022)	-0.11 (p = 0.0026)	-0.14 (p = 0.0067)
	800	0.0055 (p = 0.92)	-0.091 (p = 0.22)	-0.041 (p = 0.44)	-0.019 (p = 0.77)	-0.14 (p = 0.0045)	-0.055 (p = 0.52)	-0.11 (p = 0.023)	-0.051 (p = 0.50)
**Weighted endemism**									
	50	-0.026 (p = 0.013)	-0.025 (p = 0.027)	-0.047 (p = 0)	-0.044 (p = 0)	-0.023 (p = 0.019)	-0.044 (p = 0.0003)	-0.032 (p = 0.0009)	-0.036 (p = 0.0018)
	100	-0.0239 (p = 0.063)	-0.028 (p = 0.034)	-0.034 (p = 0.0064)	-0.021 (p = 0.11)	-0.018 (p = 0.17)	-0.036 (p = 0.012)	-0.036 (p = 0.0028)	-0.041 (p = 0.001)
	200	-0.0203 (p = 0.31)	-0.023 (p = 0.25)	-0.035 (p = 0.055)	-0.018 (p = 0.33)	-0.012 (p = 0.56)	-0.029 (p = 0.15)	-0.037 (p = 0.055)	-0.04 (p = 0.027)
	400	-0.0096 (p = 0.78)	-0.011 (p = 0.74)	-0.025 (p = 0.45)	-0.012 (p = 0.69)	-0.0023 (p = 0.94)	-0.032 (p = 0.28)	-0.027 (p = 0.43)	-0.033 (p = 0.28)
	800	0.049 (p = 0.39)	0.0067 (p = 0.9)	-0.018 (p = 0.72)	0.038 (p = 0.47)	0.022 (p = 0.66)	0.0071 (p = 0.89)	0.014 (p = 0.78)	-0.043 (p = 0.47)
**Phylogenetic endemism**									
	50	-0.067 (p = 0)	-0.067 (p = 0)	-0.085 (p = 0)	-0.075 (p = 0)	-0.082 (p = 0)	-0.086 (p = 0)	-0.10 (p = 0)	-0.08 (p = 0)
	100	-0.0727 (p = 0)	-0.073 (p = 0)	-0.095 (p = 0)	-0.081 (p = 0)	-0.084 (p = 0)	-0.099 (p = 0)	-0.10 (p = 0)	-0.099 (p = 0)
	200	-0.064 (p = 0.0002)	-0.069 (p = 0.0001)	-0.095 (p = 0)	-0.075 (p = 0)	-0.087 (p = 0)	-0.11 (p = 0)	-0.11 (p = 0)	-0.077 (p = 0)
	400	-0.068 (p = 0.022)	-0.088 (p = 0.0051)	-0.11 (p = 0.0014)	-0.076 (p = 0.01)	-0.089 (p = 0.0019)	-0.14 (p = 0.0001)	-0.14 (p = 0)	-0.095 (p = 0.0006)
	800	-0.035 (p = 0.5)	-0.10 (p = 0.074)	-0.11 (p = 0.032)	-0.045 (p = 0.44)	-0.09 (p = 0.063)	-0.11 (p = 0.047)	-0.13 (p = 0.011)	-0.079 (p = 0.11)
**Beta diversity**									
	50	-0.036 (p = 0.0001)	-0.045 (p = 0)	-0.036 (p = 0)	-0.052 (p = 0.0001)	-0.093 (p = 0)	-0.042 (p = 0)	-0.063 (p = 0)	-0.045 (p = 0)
	100	-0.14 (p = 0)	-0.08 (p = 0.0001)	-0.11 (p = 0)	-0.12 (p = 0)	-0.095 (p = 0)	-0.066 (p = 0.0003)	-0.057 (p = 0)	-0.046 (p = 0)
	200	-0.19 (p = 0)	-0.17 (p = 0)	-0.21 (p = 0)	-0.17 (p = 0)	-0.12 (p = 0)	-0.24 (p = 0)	-0.2 (p = 0)	-0.15 (p = 0)
	400	-0.15 (p = 0.0044)	-0.2 (p = 0.0007)	-0.098 (p = 0.022)	-0.095 (p = 0.0062)	-0.068 (p = 0.077)	-0.14 (p = 0.0027)	-0.14 (p = 0.0033)	-0.17 (p = 0.0015)
	800	-0.13 (p = 0.072)	-0.12 (p = 0.14)	-0.23 (p = 0.0053)	-0.10 (p = 0.098)	-0.19 (p = 0.0046)	-0.24 (p = 0.0026)	-0.19 (p = 0.0022)	-0.06 (p = 0.45)
**Phylogenetic beta diversity**									
	50	-0.42 (p = 0)	-0.51 (p = 0)	-0.36 (p = 0)	-0.47 (p = 0)	-0.46 (p = 0)	-0.47 (p = 0)	-0.39 (p = 0)	-0.52 (p = 0)
	100	-0.45 (p = 0)	-0.55 (p = 0)	-0.39 (p = 0)	-0.50 (p = 0)	-0.48 (p = 0)	-0.51 (p = 0)	-0.44 (p = 0)	-0.56 (p = 0)
	200	-0.46 (p = 0)	-0.57 (p = 0)	-0.41 (p = 0)	-0.54 (p = 0)	-0.49 (p = 0)	-0.56 (p = 0)	-0.52 (p = 0)	-0.6 (p = 0)
	400	-0.47 (p = 0)	-0.59 (p = 0)	-0.41 (p = 0)	-0.55 (p = 0)	-0.49 (p = 0)	-0.56 (p = 0)	-0.54 (p = 0)	-0.62 (p = 0)
	800	0.0055 (p = 0.93)	-0.14 (p = 0)	-0.1201 (p = 0.047)	0.0097 (p = 0.88)	-0.097 (p = 0.11)	-0.077 (p = 0.21)	-0.029 (p = 0.64)	-0.26 (p = 0)

Supplementary Table 3. Overlap of future unprotected hotspots of species richness with marine ecoregions of the world. The number of unprotected cells in each marine ecoregion is also indicated in parentheses.

RCP	T1 (2040-2050)	T2 (2090-2100)
**2.6**	East China Sea (10), Great Australian Bight (5), Southern China (4), Houtman (2), Southeast Papua New Guinea (2), Leeuwin (1), South China Sea Oceanic Islands (1)	South China Sea Oceanic Islands (25), East China Sea (20), Gulf of Tonkin (17), Southern China (16), Southern Vietnam (9), Great Australian Bight (7), Sunda Shelf/Java Sea (7), Northern Bay of Bengal (5), South Kuroshio (4), Andaman Sea Coral Coast (3), Southeast Papua New Guinea (3), Andaman and Nicobar Islands (2), Central Kuroshio Current (2), Gulf of Papua (2), Houtman (1), Leeuwin (1), Sea of Japan/East Sea (1), Torres Strait Northern Great Barrier Reef (1)
**4.5**	East China Sea (4), Great Australian Bight (3), Southeast Papua New Guinea (2), Houtman (1), Leeuwin (1), Solomon Sea (1)	East China Sea (11), Southern China (8), Great Australian Bight (4), South China Sea Oceanic Islands (3), Central Kuroshio Current (1), Gulf of Tonkin (1), Houtman (1), Leeuwin (1)
**6.0**	East China Sea (11), Great Australian Bight (3), Southeast Papua New Guinea (2), Houtman (1), Leeuwin (1), South China Sea Oceanic Islands (1), Southern China (1)	Southeast Papua New Guinea (6), Great Australian Bight (4), East China Sea (3), Solomon Sea (2), Gulf of Papua (1), Houtman (1), Leeuwin (1)
**8.5**	East China Sea (16), Great Australian Bight (7), Southern China (5), South China Sea Oceanic Islands (3), Gulf of Tonkin (1), Houtman (1), Leeuwin (1), South Kuroshio (1)	East China Sea (6), Southeast Papua New Guinea (6), Great Australian Bight (3), Solomon Sea (2), Gulf of Papua (1), Houtman (1), Leeuwin (1)

Supplementary Table 4. Overlap of future unprotected hotspots of phylogenetic diversity with marine ecoregions of the world. The number of unprotected cells in each marine ecoregion is also indicated in parentheses.

RCP	T1 (2040-2050)	T2 (2090-2100)
**2.6**	Gulf of Tonkin (31), South China Sea Oceanic Islands (29), Southern China (21), Southern Vietnam (10), South Kuroshio (8), Southeast Papua New Guinea (7), Sunda Shelf/Java Sea (6), East China Sea (5), Gulf of Papua (5), Solomon Sea (4), Ningaloo (1), Shark Bay (1), Torres Strait Northern Great Barrier Reef (1)	South China Sea Oceanic Islands (36), Gulf of Tonkin (27), Southern China (23), Southern Vietnam (10), South Kuroshio (7), Sunda Shelf/Java Sea (7), Southeast Papua New Guinea (6), East China Sea (5), Gulf of Papua (3), Solomon Sea (3), Houtman (1), Ningaloo (1), Shark Bay (1), Torres Strait Northern Great Barrier Reef (1)
**4.5**	South China Sea Oceanic Islands (30), Gulf of Tonkin (27), Southern China (18), Gulf of Papua (4), Southern Vietnam (3), Sunda Shelf/Java Sea (3), Solomon Sea (2), South Kuroshio (2), Southeast Papua New Guinea (2), East China Sea (1), Ningaloo (1), Shark Bay (1), Torres Strait Northern Great Barrier Reef (1)	Gulf of Tonkin (24), South China Sea Oceanic Islands (18), Southern China (16), East China Sea (5), Southern Vietnam (3), Gulf of Papua (1), Ningaloo (1), Shark Bay (1)
**6.0**	South China Sea Oceanic Islands (20), Gulf of Tonkin (16), Southern China (10), Southern Vietnam (8), Sunda Shelf/Java Sea (7), East China Sea (6), Southeast Papua New Guinea (5), Gulf of Papua (3), Solomon Sea (2), South Kuroshio (1), Torres Strait Northern Great Barrier Reef (1)	South China Sea Oceanic Islands (13), Southern China (11), Gulf of Tonkin (10), Southeast Papua New Guinea (7), Southern Vietnam (6), Solomon Sea (4), Sunda Shelf/Java Sea (4), Gulf of Papua (3), South Kuroshio (2), Ningaloo (1), Shark Bay (1), Torres Strait Northern Great Barrier Reef (1)
**8.5**	South China Sea Oceanic Islands (32), Gulf of Tonkin (23), Southern China (16), East China Sea (9), Southern Vietnam (9), South Kuroshio (7), Southeast Papua New Guinea (7), Sunda Shelf/Java Sea (7), Solomon Sea (4), Gulf of Papua (3), Houtman (1), Torres Strait Northern Great Barrier Reef (1)	South China Sea Oceanic Islands (29), Gulf of Tonkin (24), Southern China (14), Southern Vietnam (11), Southeast Papua New Guinea (8), Sunda Shelf/Java Sea (8), Gulf of Papua (4), Solomon Sea (4), East China Sea (3), South Kuroshio (3), Shark Bay (2), Houtman (1), Ningaloo (1), Torres Strait Northern Great Barrier Reef (1)

Supplementary Table 5. Overlap of future unprotected hotspots of weighted endemism with marine ecoregions of the world. The number of unprotected cells in each marine ecoregion is also indicated in parentheses.

RCP	T1 (2040-2050)	T2 (2090-2100)
**2.6**	Great Australian Bight (17), Yellow Sea (17), Sulawesi Sea/Makassar Strait (10), Bermuda (9), East China Sea (5), Houtman (4), Leeuwin (4), Cape Howe (3), Western Bassian (3), Bassian (2), Shark Bay (2), Sea of Japan/East Sea (1), South Australian Gulfs (1)	Great Australian Bight (17), Yellow Sea (15), Bermuda (9), Sulawesi Sea/Makassar Strait (7), East China Sea (5), Houtman (5), Leeuwin (5), Cape Howe (3), Shark Bay (3), Western Bassian (3), Bassian (2), Sea of Japan/East Sea (2), South Australian Gulfs (2), Ningaloo (1)
**4.5**	Great Australian Bight (16), Yellow Sea (14), Bermuda (9), Sulawesi Sea/Makassar Strait (9), Houtman (4), Leeuwin (4), East China Sea (3), Western Bassian (3), Bassian (2), Cape Howe (1), Sea of Japan/East Sea (1), Shark Bay (1), South Australian Gulfs (1)	Great Australian Bight (18), Yellow Sea (16), Sulawesi Sea/Makassar Strait (10), Bermuda (9), East China Sea (6), Houtman (5), Leeuwin (5), Western Bassian (4), Cape Howe (3), Shark Bay (3), South Australian Gulfs (3), Sea of Japan/East Sea (2), Bassian (1), Ningaloo (1)
**6.0**	Great Australian Bight (18), Sulawesi Sea/Makassar Strait (10), Bermuda (9), Houtman (4), Leeuwin (4), Cape Howe (3), Western Bassian (3), Yellow Sea (3), Shark Bay (2), Bassian (1), East China Sea (1), Sea of Japan/East Sea (1), South Australian Gulfs (1)	Great Australian Bight (17), Yellow Sea (13), Sulawesi Sea/Makassar Strait (11), Bermuda (8), Houtman (5), Leeuwin (5), Western Bassian (3), Bassian (2), East China Sea (2), Shark Bay (2), South Australian Gulfs (2), Palawan/North Borneo (1)
**8.5**	Yellow Sea (19), Great Australian Bight (16), Sulawesi Sea/Makassar Strait (10), Bermuda (9), East China Sea (8), Houtman (4), Leeuwin (4), Cape Howe (3), Western Bassian (3), Sea of Japan/East Sea (2), Bassian (1), Shark Bay (1), South Australian Gulfs (1)	Great Australian Bight (16), Sulawesi Sea/Makassar Strait (15), Bermuda (8), Houtman (4), Leeuwin (4), Bassian (3), Cape Howe (3), East China Sea (2), Sea of Japan/East Sea (2), Western Bassian (2), Palawan/North Borneo (1), Shark Bay (1), South Australian Gulfs (1)

Supplementary Table 6. Overlap of future unprotected hotspots of phylogenetic endemism with marine ecoregions of the world. The number of unprotected cells in each marine ecoregion is also indicated in parentheses.

RCP	T1 (2040-2050)	T2 (2090-2100)
**2.6**	Great Australian Bight (17), Yellow Sea (17), Sulawesi Sea/Makassar Strait (10), Bermuda (9), East China Sea (5), Leeuwin (5), Houtman (4), Western Bassian (4), Bassian (2), South Australian Gulfs (2), Manning-Hawkesbury (1), Ningaloo (1), Sea of Japan/East Sea (1), Shark Bay (1)	Great Australian Bight (17), Yellow Sea (15), Bermuda (9), Sulawesi Sea/Makassar Strait (7), East China Sea (5), Leeuwin (5), Western Bassian (5), Houtman (4), South Australian Gulfs (3), Sea of Japan/East Sea (2), Bassian (1), Cape Howe (1), Manning-Hawkesbury (1), Ningaloo (1), Shark Bay (1)
**4.5**	Great Australian Bight (17), Yellow Sea (14), Bermuda (9), Sulawesi Sea/Makassar Strait (9), Leeuwin (4), Lord Howe and Norfolk Islands (4), Western Bassian (4), Cape Howe (3), East China Sea (3), Houtman (3), Bassian (2), Shark Bay (2), South Australian Gulfs (2), Manning-Hawkesbury (1), Ningaloo (1), Sea of Japan/East Sea (1)	Great Australian Bight (17), Yellow Sea (15), Sulawesi Sea/Makassar Strait (10), Bermuda (9), Leeuwin (5), Houtman (4), Western Bassian (4), Cape Howe (3), South Australian Gulfs (3), Bassian (2), East China Sea (2), Sea of Japan/East Sea (2), Shark Bay (2), Manning-Hawkesbury (1), Ningaloo (1)
**6.0**	Great Australian Bight (19), Sulawesi Sea/Makassar Strait (10), Bermuda (9), Leeuwin (5), Houtman (4), Western Bassian (4), Cape Howe (3), South Australian Gulfs (3), Yellow Sea (3), Bassian (1), East China Sea (1), Sea of Japan/East Sea (1)	Great Australian Bight (16), Yellow Sea (13), Sulawesi Sea/Makassar Strait (11), Bermuda (8), Leeuwin (5), Houtman (4), Western Bassian (4), South Australian Gulfs (3), Bassian (2), East China Sea (2), Manning-Hawkesbury (1), Palawan/North Borneo (1)
**8.5**	Great Australian Bight (17), Sulawesi Sea/Makassar Strait (10), Bermuda (9), East China Sea (5), Leeuwin (5), Houtman (4), Western Bassian (4), South Australian Gulfs (3), Sea of Japan/East Sea (2), Bassian (1), Cape Howe (1), Ningaloo (1), Shark Bay (1), Yellow Sea (1)	Great Australian Bight (16), Sulawesi Sea/Makassar Strait (15), Bermuda (8), Leeuwin (6), Western Bassian (5), Bassian (4), Cape Howe (3), Houtman (3), South Australian Gulfs (3), Manning-Hawkesbury (1), Palawan/North Borneo (1)

Supplementary Table 7. Overlap of future unprotected hotspots of beta diversity with marine ecoregions of the world. The number of unprotected cells in each marine ecoregion is also indicated in parentheses.

RCP	T1 (2040-2050)	T2 (2090-2100)
**2.6**	Cape Verde (31), Hawaii (21), Lord Howe and Norfolk Islands (17), Southern California Bight (17), Southeastern Brazil (8), Bermuda (7), Magdalena Transition (7), Mexican Tropical Pacific (6), Northern Gulf of Mexico (4), Oyashio Current (4), Cortezian (3), Guayaquil (2), Gulf of Alaska (2), North and East Barents Sea (2), Northern California (2), Oregon, Washington, Vancouver Coast and Shelf (2), Revillagigedos (2), Sahelian Upwelling (2), South Kuroshio (2), Southern Gulf of Mexico (2), Bahamian (1), Central Chile (1), East China Sea (1), Northern and Central Red Sea (1), Ogasawara Islands (1), Sea of Okhotsk (1), Southeast Madagascar (1), Vanuatu (1)	Hawaii (41), Cape Verde (22), Southern California Bight (13), Lord Howe and Norfolk Islands (9), Bermuda (7), Mexican Tropical Pacific (7), Magdalena Transition (6), Guayaquil (3), Revillagigedos (3), Tweed-Moreton (3), Cortezian (2), Manning-Hawkesbury (2), Northern Gulf of Mexico (2), Sahelian Upwelling (2), Southern Gulf of Mexico (2), Central Chile (1), Central Peru (1), Ogasawara Islands (1), South Kuroshio (1)
**4.5**	Cape Verde (23), Hawaii (16), Southern California Bight (11), Lord Howe and Norfolk Islands (10), Magdalena Transition (9), Bermuda (8), Northern Gulf of Mexico (8), Southeastern Brazil (8), Mexican Tropical Pacific (7), Revillagigedos (6), Cortezian (3), Guayaquil (3), North and East Barents Sea (3), Southern Gulf of Mexico (3), Central Kuroshio Current (2), Manning-Hawkesbury (2), Oyashio Current (2), Sahelian Upwelling (2), South Kuroshio (2), Carolinian (1), Central Chile (1), Central Peru (1), East China Sea (1), Eastern Brazil (1), Gilbert/Ellis Islands (1), Juan Fernandez and Desventuradas (1), Ogasawara Islands (1)	Cape Verde (29), Southern California Bight (24), Hawaii (21), Magdalena Transition (14), Northern Gulf of Mexico (14), Lord Howe and Norfolk Islands (13), Southeastern Brazil (11), Bermuda (8), Mexican Tropical Pacific (8), Revillagigedos (7), Central Kuroshio Current (6), Southern Gulf of Mexico (6), Oyashio Current (4), Cortezian (3), Guayaquil (3), Northern California (3), Oregon, Washington, Vancouver Coast and Shelf (3), Sahelian Upwelling (3), Carolinian (2), Saharan Upwelling (2), Sea of Okhotsk (2), South Kuroshio (2), Central Chile (1), Central Peru (1), East China Sea (1), Eastern Brazil (1), Floridian (1), Gilbert/Ellis Islands (1), Juan Fernandez and Desventuradas (1)
**6.0**	Cape Verde (32), Southern California Bight (24), Lord Howe and Norfolk Islands (21), Hawaii (17), Northern Gulf of Mexico (14), Magdalena Transition (11), Mexican Tropical Pacific (8), Revillagigedos (7), Southeastern Brazil (7), Southern Gulf of Mexico (6), Bermuda (5), Vanuatu (5), Cortezian (4), Guayaquil (3), Northern California (3), Sahelian Upwelling (3), Carolinian (2), Levantine Sea (2), Oyashio Current (2), Sea of Okhotsk (2), Central Kuroshio Current (1), Central Peru (1), Eastern Brazil (1), Gilbert/Ellis Islands (1), Gulf of Alaska (1), North and East Barents Sea (1), Ogasawara Islands (1), Saharan Upwelling (1), Southeast Madagascar (1), Western and Northern Madagascar (1)	Southern California Bight (31), Cape Verde (25), Hawaii (20), Lord Howe and Norfolk Islands (15), Magdalena Transition (14), Mexican Tropical Pacific (9), Northern Gulf of Mexico (9), Southeastern Brazil (9), Revillagigedos (8), Bermuda (7), Northern California (6), Oregon, Washington, Vancouver Coast and Shelf (6), Oyashio Current (6), Cortezian (5), Central Kuroshio Current (4), Southern Gulf of Mexico (4), Guayaquil (3), Sea of Okhotsk (3), South Kuroshio (3), Manning-Hawkesbury (2), Sahelian Upwelling (2), Bassian (1), Carolinian (1), Central Chile (1), Central Peru (1), East China Sea (1), Eastern Brazil (1), Floridian (1), Gilbert/Ellis Islands (1), Ogasawara Islands (1), Samoa Islands (1), Tweed-Moreton (1)
**8.5**	Hawaii (23), Southern California Bight (22), Cape Verde (18), Lord Howe and Norfolk Islands (14), Magdalena Transition (14), Mexican Tropical Pacific (9), North and East Barents Sea (7), Bermuda (6), Oyashio Current (5), Revillagigedos (5), Sahelian Upwelling (4), Cortezian (3), Northern Gulf of Mexico (3), Oregon, Washington, Vancouver Coast and Shelf (3), Southeastern Brazil (3), Tweed-Moreton (3), Vanuatu (3), Guayaquil (2), Juan Fernandez and Desventuradas (2), Phoenix/Tokelau/Northern Cook Islands (2), Samoa Islands (2), Sea of Okhotsk (2), Southern Gulf of Mexico (2), Araucanian (1), Eastern Brazil (1), Gulf of Alaska (1), Manning-Hawkesbury (1), Northern California (1), Ogasawara Islands (1), Saharan Upwelling (1)	Hawaii (28), Southern California Bight (14), Cape Verde (13), Southeastern Brazil (11), Lord Howe and Norfolk Islands (10), Magdalena Transition (10), Bermuda (7), Mexican Tropical Pacific (7), Revillagigedos (6), Northern Gulf of Mexico (5), Cortezian (3), Guayaquil (3), Southern Gulf of Mexico (3), Vanuatu (3), Carolinian (2), Central Kuroshio Current (2), Araucanian (1), Azores Canaries Madeira (1), Central Chile (1), Central Peru (1), Eastern Brazil (1), Gilbert/Ellis Islands (1), Juan Fernandez and Desventuradas (1), Ogasawara Islands (1), South Kuroshio (1), Tweed-Moreton (1), Western and Northern Madagascar (1)

Supplementary Table 8. Overlap of future unprotected hotspots of phylogenetic beta diversity with marine ecoregions of the world. The number of unprotected cells in each marine ecoregion is also indicated in parentheses.

RCP	T1 (2040-2050)	T2 (2090-2100)
**2.6**	Cape Verde (31), Lord Howe and Norfolk Islands (20), Southeastern Brazil (8), Gilbert/Ellis Islands (7), Mexican Tropical Pacific (6), Marshall Islands (5), Northern Gulf of Mexico (4), Cortezian (3), Central Kuroshio Current (2), Guayaquil (2), Magdalena Transition (2), North and East Barents Sea (2), Revillagigedos (2), Sahelian Upwelling (2), South Kuroshio (2), Southern Gulf of Mexico (2), Bahamian (1), Central Chile (1), Hawaii (1), Levantine Sea (1), Natal (1), Ogasawara Islands (1), Southeast Madagascar (1)	Cape Verde (22), Hawaii (17), Gilbert/Ellis Islands (13), Lord Howe and Norfolk Islands (10), Mexican Tropical Pacific (8), Cortezian (3), Guayaquil (3), Phoenix/Tokelau/Northern Cook Islands (3), Revillagigedos (3), Magdalena Transition (2), Northern Gulf of Mexico (2), Sahelian Upwelling (2), Samoa Islands (2), Southern Cook/Austral Islands (2), Southern Gulf of Mexico (2), Central Chile (1), Central Peru (1), Ogasawara Islands (1)
**4.5**	Cape Verde (23), Lord Howe and Norfolk Islands (13), Northern Gulf of Mexico (10), Southeastern Brazil (8), Gilbert/Ellis Islands (6), Mexican Tropical Pacific (6), Revillagigedos (5), Natal (4), Cortezian (3), Guayaquil (3), North and East Barents Sea (3), Southern Gulf of Mexico (3), Magdalena Transition (2), Sahelian Upwelling (2), Carolinian (1), Central Chile (1), Central Peru (1), Eastern Brazil (1), Juan Fernandez and Desventuradas (1), Levantine Sea (1), Marshall Islands (1), Ogasawara Islands (1)	Cape Verde (29), Northern Gulf of Mexico (14), Lord Howe and Norfolk Islands (13), Southeastern Brazil (11), Mexican Tropical Pacific (7), Revillagigedos (6), Southern Gulf of Mexico (6), Gilbert/Ellis Islands (4), Cortezian (3), Guayaquil (3), Sahelian Upwelling (3), Carolinian (2), Magdalena Transition (2), Saharan Upwelling (2), Agulhas Bank (1), Central Chile (1), Central Kuroshio Current (1), Central Peru (1), Eastern Brazil (1), Floridian (1), Juan Fernandez and Desventuradas (1), Levantine Sea (1), South Kuroshio (1)
**6.0**	Cape Verde (32), Lord Howe and Norfolk Islands (21), Northern Gulf of Mexico (16), Levantine Sea (8), Mexican Tropical Pacific (7), Southeastern Brazil (7), Revillagigedos (6), Southern Gulf of Mexico (6), Cortezian (4), Guayaquil (3), Sahelian Upwelling (3), Carolinian (2), Magdalena Transition (2), Phoenix/Tokelau/Northern Cook Islands (2), Samoa Islands (2), Central Kuroshio Current (1), Central Peru (1), Eastern Brazil (1), North and East Barents Sea (1), Saharan Upwelling (1), Southeast Madagascar (1), Western and Northern Madagascar (1)	Cape Verde (25), Lord Howe and Norfolk Islands (15), Northern Gulf of Mexico (10), Levantine Sea (9), Southeastern Brazil (9), Mexican Tropical Pacific (8), Revillagigedos (7), Cortezian (5), Gilbert/Ellis Islands (5), North and East Barents Sea (4), Southern Gulf of Mexico (4), Central Kuroshio Current (3), Guayaquil (3), Magdalena Transition (2), Sahelian Upwelling (2), Bassian (1), Carolinian (1), Central Chile (1), Central Peru (1), Eastern Brazil (1), Floridian (1), Samoa Islands (1), South Kuroshio (1), Tweed-Moreton (1)
**8.5**	Cape Verde (18), Lord Howe and Norfolk Islands (18), Mexican Tropical Pacific (7), North and East Barents Sea (7), Phoenix/Tokelau/Northern Cook Islands (6), Sahelian Upwelling (4), Samoa Islands (4), Northern Gulf of Mexico (3), Revillagigedos (3), Southeastern Brazil (3), Cortezian (2), Guayaquil (2), Juan Fernandez and Desventuradas (2), Magdalena Transition (2), Southern Gulf of Mexico (2), Araucanian (1), Eastern Brazil (1), Gilbert/Ellis Islands (1), Levantine Sea (1), Ogasawara Islands (1), Saharan Upwelling (1)	Lord Howe and Norfolk Islands (14), Cape Verde (13), Southeastern Brazil (11), Mexican Tropical Pacific (6), Northern Gulf of Mexico (5), Revillagigedos (5), Phoenix/Tokelau/Northern Cook Islands (4), Cortezian (3), Gilbert/Ellis Islands (3), Guayaquil (3), Southern Gulf of Mexico (3), Carolinian (2), Hawaii (2), Magdalena Transition (2), Southern Cook/Austral Islands (2), Araucanian (1), Azores Canaries Madeira (1), Central Chile (1), Central Peru (1), Eastern Brazil (1), Juan Fernandez and Desventuradas (1), Ogasawara Islands (1), Saharan Upwelling (1), Tweed-Moreton (1), Western and Northern Madagascar (1)
