# Supplementary material for: Reorganization of seagrass communities in a changing climate
Source: Nat Plants. 2023 Jun 19;9(7):1034–43. doi: 10.1038/s41477-023-01445-6 (PMC10356593; doi:10.1038/s41477-023-01445-6)
Supplement: Supplementary file 2 — Reporting Summary [file 41477_2023_1445_MOESM2_ESM.pdf]

nature portfolio

Corresponding author(s): BARNABAS H. DARU

Last updated by author(s): May 13, 2023

Reporting Summary

Nature Portfolio wishes to improve the reproducibility of the work that we publish. This form provides structure for consistency and transparency in reporting. For further information on Nature Portfolio policies, see our Editorial Policies and the Editorial Policy Checklist.

Statistics

For all statistical analyses, confirm that the following items are present in the figure legend, table legend, main text, or Methods section.

n/a	Confirmed	
☐	☒	The exact sample size (*n*) for each experimental group/condition, given as a discrete number and unit of measurement
☐	☒	A statement on whether measurements were taken from distinct samples or whether the same sample was measured repeatedly
☒	☐	The statistical test(s) used AND whether they are one- or two-sided<br>*Only common tests should be described solely by name; describe more complex techniques in the Methods section.*
☐	☒	A description of all covariates tested
☐	☒	A description of any assumptions or corrections, such as tests of normality and adjustment for multiple comparisons
☐	☒	A full description of the statistical parameters including central tendency (e.g. means) or other basic estimates (e.g. regression coefficient) AND variation (e.g. standard deviation) or associated estimates of uncertainty (e.g. confidence intervals)
☐	☒	For null hypothesis testing, the test statistic (e.g. *F*, *t*, *r*) with confidence intervals, effect sizes, degrees of freedom and *P* value noted<br>*Give P values as exact values whenever suitable.*
☒	☐	For Bayesian analysis, information on the choice of priors and Markov chain Monte Carlo settings
☒	☐	For hierarchical and complex designs, identification of the appropriate level for tests and full reporting of outcomes
☒	☐	Estimates of effect sizes (e.g. Cohen's *d*, Pearson's *r*), indicating how they were calculated

Our web collection on statistics for biologists contains articles on many of the points above.

Software and code

Policy information about availability of computer code

Data collection	The open source software used to collect the is the R computing language version 4.2.2 (2022-10-31) -- "Innocent and Trusting". The R code and data documentation necessary to repeat our analyses have been made available in the Figshare database [https://doi.org/10.6084/m9.figshare.21905826] under the folder "SCRIPTS".
Data analysis	The following open source software and R packages were utilized for analyzing the data: R v.4.2.2 and packages phyloregion v.1.0.8, terra v.1.7-3, ape v.5.6-2, spatialreg v.1.2-6, ggplot2 v.3.4.0, adephylo v.1.1-13, phytools v.1.2-0, scico v.1.3.0, and ggtree v.3.4.1. Custom R scripts were developed to analyze the spatial distribution data, which are available permanently at the Figshare database [https://doi.org/10.6084/m9.figshare.21905826].

For manuscripts utilizing custom algorithms or software that are central to the research but not yet described in published literature, software must be made available to editors and reviewers. We strongly encourage code deposition in a community repository (e.g. GitHub). See the Nature Portfolio guidelines for submitting code & software for further information.

Data

Policy information about availability of data

All manuscripts must include a data availability statement. This statement should provide the following information, where applicable:

- Accession codes, unique identifiers, or web links for publicly available datasets
- A description of any restrictions on data availability
- For clinical datasets or third party data, please ensure that the statement adheres to our policy

The range polygons downloaded from the International Union for the Conservation of Nature (IUCN) are available for download at Figshare (https://doi.org/10.6084/m9.figshare.21905826). The datasets, phylogeny, vector polygons, and R codes used for the analysis are also archived on Figshare (https://doi.org/10.6084/m9.figshare.21905826). The R scripts were developed using open source software including R v.4.2.2 and packages phyloregion v.1.0.8, terra v.1.7-3, ape 5.6-2, spatialreg v.1.2-6, ggplot2 v.3.4.0, adephylo v.1.1-13, phytools v.1.2-0, scico 1.3.0, and ggtree 3.4.1. Custom R scripts used to analyze the spatial distribution data are also permanently available at the Figshare database (https://doi.org/10.6084/m9.figshare.21905826).

Research involving human participants, their data, or biological material

Policy information about studies with human participants or human data. See also policy information about sex, gender (identity/presentation), and sexual orientation and race, ethnicity and racism.

Reporting on sex and gender	Not applicable in this study
Reporting on race, ethnicity, or other socially relevant groupings	Not applicable in this study
Population characteristics	Not applicable in this study
Recruitment	Not applicable in this study
Ethics oversight	Not applicable in this study

Note that full information on the approval of the study protocol must also be provided in the manuscript.

Field-specific reporting

Please select the one below that is the best fit for your research. If you are not sure, read the appropriate sections before making your selection.

☐ Life sciences ☐ Behavioural & social sciences ☒ Ecological, evolutionary & environmental sciences

For a reference copy of the document with all sections, see nature.com/documents/nr-reporting-summary-flat.pdf

Ecological, evolutionary & environmental sciences study design

All studies must disclose on these points even when the disclosure is negative.

Study description	This study analyzes how different facets of seagrass diversity will respond to future climate change scenarios across the globe and compare their coverage with the existing network of marine protected areas. Geographic data were compiled from GBIF and IUCN, current and global future climate layers were sourced from the Bio-ORACLE v.2.2 database, phylogenetic data from Daru et al. Biol. Conserv. 213, 351-356 (2017), and protected areas from the World Database of Protected Areas (MPAs). The data were analyzed to explore how different facets of locally distinctive α-diversity and β-diversity (changes in spatial composition) of seagrasses will respond to future climate change scenarios across the globe and contrast their coverage with the existing network of marine protected areas.
Research sample	For this study, a species record was considered as a research sample. We first collated occurrence data of seagrasses from public open-source databases: the Global Biodiversity Information Facility (GBIF; https://www.gbif.org/), Seagrass Watch (www.seagrasswatch.org), iDigBio (https://www.idigbio.org/), Ocean Biogeographic Information System (OBIS; https://obis.org/), and the International Union of Conservation of Nature (IUCN). However, we previously demonstrated that occurrence records of seagrasses are very scarce and prevalent with coverage gaps and biases, and thus can constrain research to assess present and future seagrass response from climate change. Therefore, we used range polygons from the IUCN to model present and future distributions of seagrasses under climate scenarios. The final checklist included 66 valid species which were all included in the analysis.
Sampling strategy	We converted the species polygons into raster format at a grain resolution of 5 arcmin (~9.2 km), and then to points and treat them as real point occurrence records. For each species, we spatially thinned occurrences to 500 records to avoid spatial bias in the modeling. We then standardized the taxonomy of each seagrass species by checking for misspellings, synonyms, formatting errors, hybrid names, and infraspecific ranks, against the backbone taxonomy from the World Flora Online v.2022.05. Taxonomic harmonization was done using the R package WorldFlora and manually checked in cases of misspellings or errors.

Data collection	Data on species occurrences and distributions were collected primarily from GBIF, IUCN, and Bio-ORACLE v.2.2 by Brianna M. Rock and Barnabas H. Daru. All analyses were carried out by Barnabas H. Daru.
Timing and spatial scale	The spatial extent is global whereas the spatial grain varies along 50, 100,200,400, and 800 km spatial resolutions. Analyses of beta diversity were performed at varying extents using cluster analysis of grid cells within Marine Ecoregions of the World.<br>The timing is contemporary times corresponding to modern-day and future scenarios corresponding to climate projections in years 2050 and 2100.
Data exclusions	No data were excluded
Reproducibility	All scripts, codes, and data documentation necessary to repeat and reproduce our analyses have been made available in the Figshare database [https://doi.org/10.6084/m9.figshare.21905826] under the folder "SCRIPTS".
Randomization	Not applicable
Blinding	Blinding was not relevant in this study.

Did the study involve field work? ☐ Yes ☒ No

Reporting for specific materials, systems and methods

We require information from authors about some types of materials, experimental systems and methods used in many studies. Here, indicate whether each material, system or method listed is relevant to your study. If you are not sure if a list item applies to your research, read the appropriate section before selecting a response.

Materials & experimental systems

n/a	Involved in the study	
☒	☐	Antibodies
☒	☐	Eukaryotic cell lines
☒	☐	Palaeontology and archaeology
☒	☐	Animals and other organisms
☒	☐	Clinical data
☒	☐	Dual use research of concern
☐	☒	Plants

Methods

n/a	Involved in the study	
☒	☐	ChIP-seq
☒	☐	Flow cytometry
☒	☐	MRI-based neuroimaging

Dual use research of concern

Policy information about dual use research of concern

Hazards

Could the accidental, deliberate or reckless misuse of agents or technologies generated in the work, or the application of information presented in the manuscript, pose a threat to:

No	Yes	
☒	☐	Public health
☒	☐	National security
☒	☐	Crops and/or livestock
☒	☐	Ecosystems
☒	☐	Any other significant area

Experiments of concern

Does the work involve any of these experiments of concern:

No	Yes	
☒	☐	Demonstrate how to render a vaccine ineffective
☒	☐	Confer resistance to therapeutically useful antibiotics or antiviral agents
☒	☐	Enhance the virulence of a pathogen or render a nonpathogen virulent
☒	☐	Increase transmissibility of a pathogen
☒	☐	Alter the host range of a pathogen
☒	☐	Enable evasion of diagnostic/detection modalities
☒	☐	Enable the weaponization of a biological agent or toxin
☒	☐	Any other potentially harmful combination of experiments and agents
